# Driving Intention Recognition of Electric Wheel Loader Based on Fuzzy Control

**DOI:** 10.3390/s25010032

**Published:** 2024-12-24

**Authors:** Qihuai Chen, Yuanzheng Lin, Mingkai Xu, Haoling Ren, Guanjie Li, Tianliang Lin

**Affiliations:** 1 College of Mechanical Engineering and Automation, Huaqiao University, Xiamen 361021, China; chen.qihuai@163.com (Q.C.); lin_yuanzheng@163.com (Y.L.); 20014080067@stu.hqu.edu.cn (M.X.); happyrhlly@126.com (H.R.); li18359709827@163.com (G.L.); 2 Fujian Key Laboratory of Green Intelligent Drive and Transmission for Mobile Machinery, Xiamen 361021, China

**Keywords:** engineering machinery, wheel loader, walking, driving intention recognition, fuzzy control

## Abstract

Energy conservation and emission reduction is a common concern in various industries. The construction process of electric wheel loaders has the advantages of being zero-emission and having a high energy efficiency, and has been widely recognized by the industry. The frequent shift in wheel loader working processes poses a serious challenge to the operator. Automatic shift is an effective way to improve the operator’s comfort and safety. The driving intention is an important input judgment condition to achieve efficient automatic shift. However, the current methods of vehicle driving intention recognition mainly focus on passenger cars. The working condition of the wheel loader is significantly different from that of the passenger car, with a high shifting frequency and severe load fluctuation. The driving intention recognition method of passenger cars is difficult to transplant directly. In this paper, aiming at the characteristics of wheel loader working conditions, a fuzzy recognition method based on fuzzy control is applied to driving intention recognition for electric wheel loaders. The throttle, throttle change rate and braking signals are used as inputs for recognizing the driving intention at the current moment of the whole machine. Five types of driving intentions, namely, rapid acceleration, normal acceleration, acceleration maintenance, deceleration and braking, are defined and recognized. In order to verify the effectiveness of the proposed method, simulation and experimental research are carried out. The results show that the proposed driving intention recognition method can effectively identify the driver’s intention and provide effective shift signal input for the wheel loader.

## 1. Introduction

Energy conservation and emission reduction has become the focus of global attention. Wheel loaders account for a large proportion of construction machinery and are widely used in construction sites, mines, highways, ports and other fields. The drive of traditional fuel wheel loaders has low energy efficiency and poor emission. Electrification can effectively improve the energy efficiency and emission of the whole machine during working processes, which is one of the ideal driving schemes [1,2].

Electric loaders can be further categorized into two configurations: those that adopt a hydraulic torque converter [3,4] and those that dispense with it [5]. Among them, the efficiency of the hydraulic torque converter is relatively low due to its susceptibility to load fluctuations, significantly limiting the overall efficiency of electric drive systems [6,7]. Therefore, the current mainstream electric wheel loader uses a motor to replace the internal combustion engine, cancels the hydraulic torque converter and is directly connected to the transmission by the drive motor [8]. The multi-gear transmission is necessary to improve the power performance of the vehicle by using the large speed ratio of the low gear. By switching the gear, the drive motor works at a high efficiency and improves the economy [9,10].

The wheel loader has special working conditions and a harsh working environment. In order to ensure the power performance, it is necessary to switch gears frequently [11]. According to statistics, the wheel loader switches gears once in an average of 3.6 s (up to 1000 times per hour) [8]. It seriously increases the driver’s labor intensity and affects the safety of driving [3,12]. In addition, shifting at the wrong time cannot take advantage of the high efficiency of the electric drive, resulting in the poor economy of the vehicle. Therefore, an automatic transmission system is introduced into the electric wheel loader equipped with automatic transmission. The shift process is controlled by the controller, which can ensure the dynamic performance of the machine and take into account the economy without the influence of the driver’s operation experience. At the same time, it can also reduce the driver’s labor intensity and ensure the safety of driving.

For wheel loader driving, the driver performs a series of operations according to the loader driving state and environmental changes. The driving intention is a reflection of the state. Therefore, in the process of loader automatic shift, the driver’s control intention is an important input judgment condition to achieve efficient automatic shift. The driving intention cannot be obtained directly and needs to be obtained indirectly. Many scholars have performed a lot of research on the recognition of driving intention.

Currently, research on vehicle driving intention recognition is extensive, but it primarily focuses on passenger cars. He et al. [13] proposed a driving intention recognition and prediction model based on double-layer hidden Markov (HMM) according to the driver’s control signal and vehicle state information. The test showed that the double-layer HMM could accurately and effectively identify the driving intention and predict the driving behavior. Gebert used the visual system of a deep neural network to identify and predict the driver’s driving intention. The experimental results showed that the recognition accuracy of the model to the driver’s intention reached 83.12% [14]. Gilman et al. [15] proposed a driving perception assistance system to collect and analyze driver-related information and used real-time feedback on the driver’s intention to modify control strategies to improve the vehicle economy. Wang et al. [16] proposed a driving intention recognition model based on the random forest method for mining trucks by collecting the data of driver’s driving intention and classifying the categories of driving intention by data clustering analysis. The results showed that this method could effectively identify different driving intentions. Halim et al. [17] obtained driving characteristic data by simulating the driving environment, and used k-means clustering, the fuzzy c-means clustering algorithm and the model-based clustering algorithm to cluster the data. The three clustering algorithms all divided the data into four categories. Based on the clustering analysis results, the support vector machine of the artificial neural network and RBF kernel function was trained as a classifier to identify driving intention. The experimental results showed that the recognition accuracy of the neural network was higher than that of the support vector machine classifier.

Wang et al. classified the driver’s braking intention, selected the brake pedal opening and the brake pedal opening change rate as the input parameters of the fuzzy controller to identify the driver’s braking intention, and used the identification results for the coordinated control of the braking system. The simulation results showed that the braking control strategy with driving intention recognition effectively improved the braking energy recovery efficiency [18]. According to the characteristics of the fuel wheel loader, Yang et al. divided the driving intention into acceleration, braking and fast lifting intentions, and used the fuzzy reasoning method to identify the driving intention. The recognition results were used in the hydraulic mechanical stepless transmission control strategy. The hardware-in-the-loop simulation results showed that the control strategy reduces fuel consumption [19]. Li et al. [20] proposed a driver’s braking intention recognition based on an artificial bee colony support vector machine. After collecting and processing braking data and extracting effective eigenvalues, the recognition was carried out. The results showed that this method was more accurate and faster than the commonly used recognition methods. Liu et al. [21] collected a large number of driving samples by using the driving simulator system to carry out experiments. By comparing the differences between the lane keeping intention, lane changing intention and overtaking intention, the driving intention recognition index system was determined, and the driver intention recognition model was established by using the HMM and SVM cascade algorithm. The results showed that the recognition accuracy of the algorithm reached 95.84%, which was significantly higher than that of the HMM or SVM single algorithm. Ji et al. [22] used deep learning methods to design a long-term and short-term memory network driving intention and vehicle trajectory prediction model. The model training was completed using real data. The results show that compared with the traditional method, the driving intention recognition accuracy was higher, and the trajectory prediction accuracy was also significantly improved. Jia et al. studied the driving intention in a complex environment, proposed a driving intention game and multi-dimensional deep learning method, and established a typical driving behavior data set. The experimental results showed that the game model could identify the lane change intention, and the deep learning model predicts typical driving behavior. The method was used in the vehicle safety warning system to effectively improve the safety performance [23,24].

The research on driver intention recognition mainly focuses on passenger cars. The wheel loader is a special vehicle, and the working environment and control method are more complex and harsh than other vehicles. There are relatively few studies in this area. In general, driving intention recognition belongs to pattern recognition, and the commonly used recognition methods include artificial neural networks, hidden Markov models and fuzzy control [25]. Among them, the artificial neural network is a model established by imitating the thinking mode of human brain neurons, which does not depend on the object model and has a better nonlinear learning ability. The feature parameters or feature vectors are used as input, and a large amount of training is performed on the data samples until the output mode class meets the requirements. However, this method requires a large amount of calculation, has a long iteration time, and no semantic information in the calculation process, so it is not suitable for driver intention recognition. The hidden Markov model is a special kind of Bayesian decision model, which has a strong processing ability for time series data. Although this method can accurately identify the driver’s intention, it has high requirements for controller calculation and is not suitable for practical application on wheel loaders. Fuzzy control is a way to solve engineering problems with no boundary or ambiguity by simulating the logic of human brain thinking. The calculation of the uncertain model by fuzzy control is based on the input of the system and the fuzzy rule reasoning to obtain the output value. The driving intention is generated by the driver’s dynamic change in the driving environment and their own driving habits. There is no specific mathematical model. The fuzzy control logic fits the driver’s thinking, and the calculation amount is relatively small, which can realize real-time control.

In this paper, the electric wheel loader without the hydraulic torque converter is taken into consideration. According to the characteristics of the wheel loader working conditions, a driving intention recognition method based on fuzzy control is proposed. Based on the signals of throttle, throttle change rate and braking, the fuzzy rules of driver intention recognition are constructed to obtain the driver’s control intention of the wheel loader driving process.

## 2. Driving Intention Division and Recognition Parameter Selection

### 2.1. Driving Intention Division

**Figure 1:**
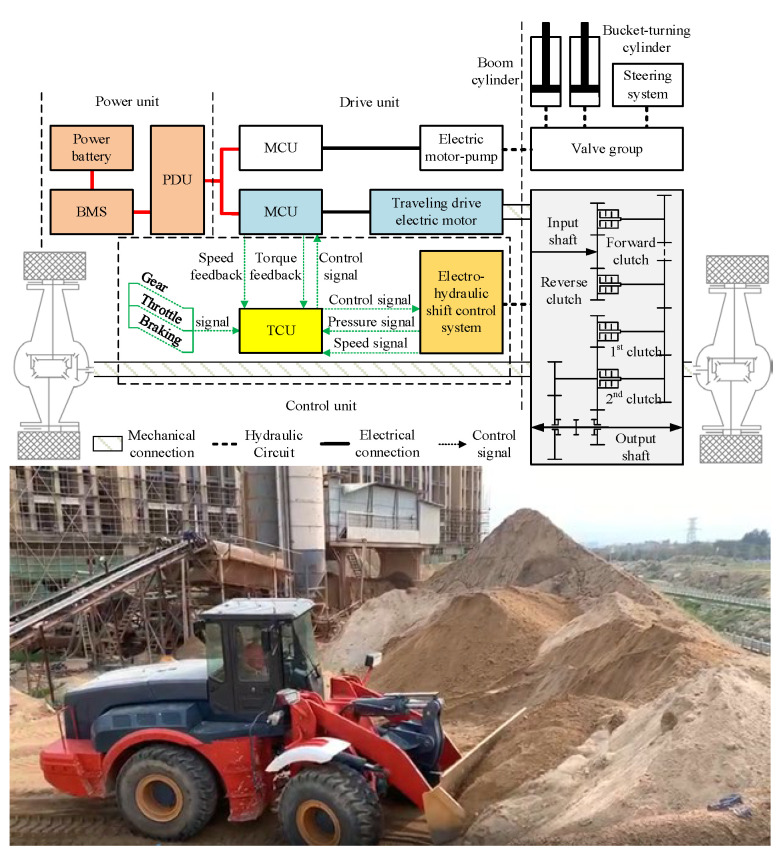
Schematic diagram of wheel loader walking and shifting.

The energy distribution scheme of the electric wheel loader is different from that of the traditional fuel wheel loader. The walking system is driven by motor and transmission. In this paper, a 5 t electric wheel loader is taken as the study’s focus. The system schematic diagram is shown in Figure 1. The intention division is closely related to the operation mode. Therefore, through the driver’s experience, the operation characteristics of the wheel loader under the working conditions are analyzed, and the driving intention of the electric wheel loader is divided into acceleration intention, deceleration intention and braking intention.

(1) Accelerated intention

The wheel loader is mainly used for shoveling and transporting materials. It has a strong periodicity and frequent start and stop under operating conditions. The wheel loader’s operation methods include ‘V’, ‘T’, ‘I’ and ‘L’, and other shapes. Among them, the ‘V’-shaped method is widely used due to its high operational efficiency and small footprint required for the work area. Therefore, taking the commonly used ‘V’-type operation mode as an example, the wheel loader has five stages in a cycle: no-load forward, shoveling, full-load backward, full-load forward, and no-load backward. In a short cycle, the number of starts is as many as four. And continuously operating in this mode, it forms a significant difference compared to the smooth working mode of a passenger car. Coupled with the large mass of the wheel loader, in order to improve the efficiency of the operation, there is often an urgent acceleration demand on the driver at the start. In the process of operation, there are often periods of fast driving to the material pile, fast retreat, fast driving to the transporter unloading, etc. At these times, the speed requirement is relatively high, and it is often necessary to remain in second gear to ensure the high speed requirement. In these cases, it is mainly based on normal acceleration and maintenance of speed. When the wheel loader is digging, due to the large resistance of the material pile, the driver usually increases the throttle pedal opening in order to complete the digging. Under the cyclic working conditions of the wheel loader, the driver’s intention demand changes dramatically. Therefore, in order to better meet the driver’s driving needs, this paper subdivides the acceleration intention of the electric wheel loader into rapid acceleration, normal acceleration and maintenance. The classification and characteristics of acceleration intention are shown in Table 1.

(2) Deceleration and braking intention

The wheel loader will frequently start and stop during cycle operation. In the case of a low urgency to reduce the speed, the driver usually reduces the throttle opening. When there is an urgent need to reduce the speed, the accelerator pedal is usually quickly loosened and the brake pedal is stepped down. In order to achieve energy saving and improve the endurance of the electric wheel loader, the corresponding regenerative braking energy recovery strategy is implemented according to the braking force during the braking process. Considering that energy recovery is not the focus of this paper, in the case of vehicle deceleration, the intention is classified as braking intention only when there is a brake pedal signal; otherwise, it is deceleration intention. In summary, the driver’s intention under the deceleration of the electric wheel loader is divided into two categories: deceleration and braking intention. The classification and characteristics of deceleration and braking intention are shown in Table 2.

### 2.2. Identification Parameter Selection

The driving intention recognition parameters of non-engineering vehicles usually select the vehicle speed, accelerator pedal opening and brake pedal opening. For wheel loaders, the vehicle speed can reflect the driver’s intention to a certain extent. However, due to the large mass of the vehicle, there will be a lag phenomenon as an identification parameter. The accelerator pedal opening can reflect the driver’s demand for vehicle acceleration, but this parameter cannot accurately reflect the driver’s urgency when accelerating, and the change rate of the accelerator pedal opening can well reflect the change speed of the accelerator pedal opening, which in turn reflects the driver’s urgency for vehicle acceleration. In addition to acceleration, the vehicle in the deceleration or braking state can also reflect the driver’s driving intention. Therefore, this paper selects three parameters of accelerator pedal opening, accelerator pedal opening change rate and brake pedal opening to identify the driving intention of electric wheel loader.

## 3. Driving Intention Recognition

### 3.1. Throttle Pedal Data Acquisition

**Figure 2:**
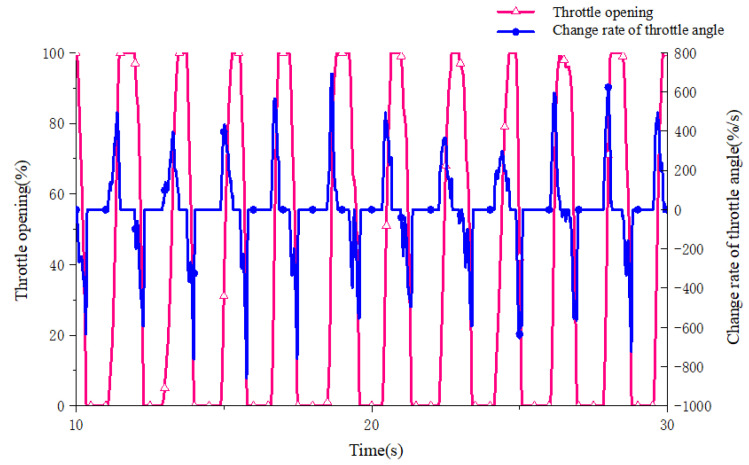
Opening and opening change rate curve under throttle fast stepping.

**Figure 3:**
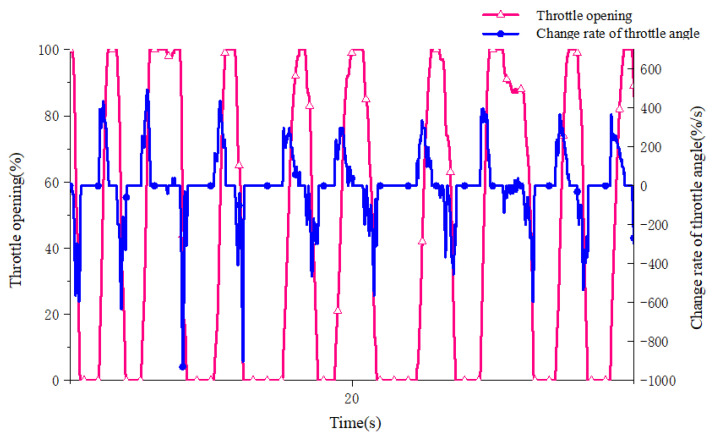
Opening and opening change rate curve under throttle quick release.

A real 5 t wheel loader is used to obtain the accelerator pedal opening, accelerator pedal opening change rate and brake pedal opening signals. An electronic accelerator pedal and brake pedal are equipped. A number of drivers with wheel loader driving experience are selected as testers, and the data of pedal opening and opening change rate under different intentions are collected and analyzed as the domain of the fuzzy controller. The limit value of the change rate of the accelerator pedal opening can only be obtained through the test. During the test, the accelerator pedal is pressed and the accelerator pedal is quickly loosened at the fastest speed many times. Some of the collected test data curves are shown in Figure 2 and Figure 3. Considering that the limit value of the throttle opening change rate needs to be left with an appropriate margin, the range of the throttle pedal opening change rate is determined to be (−1000, 800) %/s according to the test results.

### 3.2. Establishment of Fuzzy Control Input and Output Membership Function

**Figure 4:**
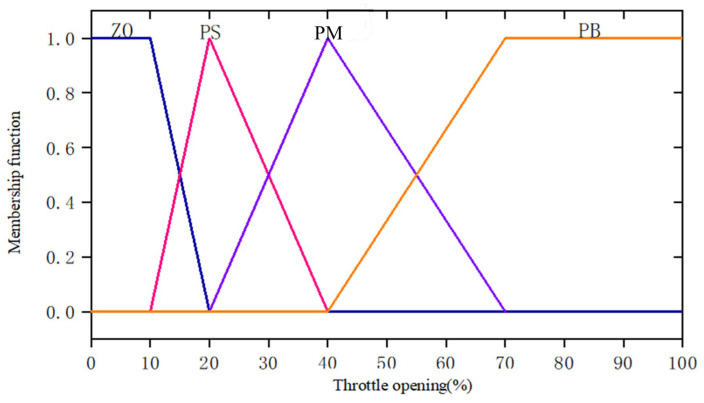
The membership function of accelerator pedal opening degree.

**Figure 5:**
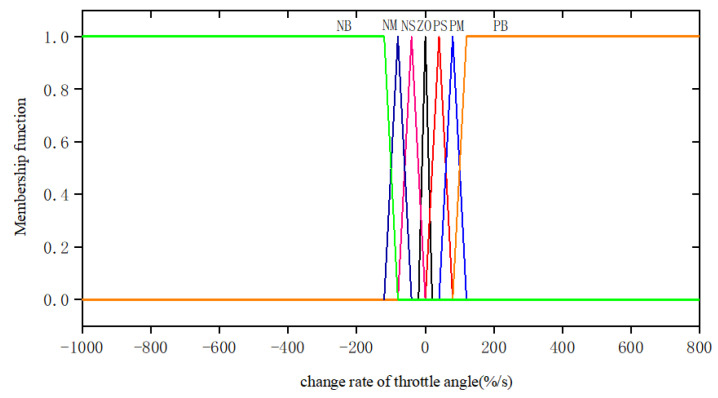
The membership function of throttle opening change rate.

The throttle pedal opening and the throttle opening change rate are used as the input of the fuzzy controller. The membership function and domain of the input variables are shown in Table 3 and Figure 4 and Figure 5.

**Figure 6:**
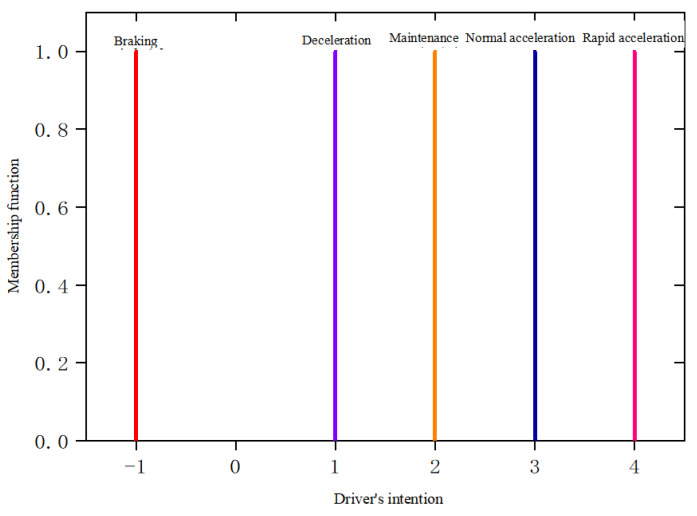
Driver’s intention membership function.

The output variable membership function and the domain membership function are shown in Table 4 and Figure 6.

### 3.3. Driving Intention Recognition Process

Firstly, the throttle change rate and throttle are used as fuzzy control inputs, and the fuzzy roadbed rule table for driver intention recognition is summarized in Table 5.

**Figure 7:**
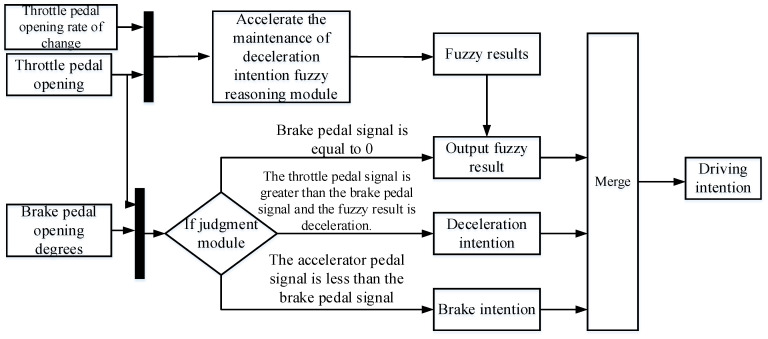
Flow chart of driving intention recognition.

The driving intention recognition result is unique at the same time, so the acceleration, deceleration and braking intention of the electric wheel loader are combined to identify this. Due to the special working conditions of the wheel loader, there may be a situation where the accelerator pedal and the brake pedal act simultaneously when the driver is operating. Therefore, the fuzzy controller is used to identify the acceleration maintenance and deceleration intention, and the braking intention and the situation of simultaneously stepping on the accelerator pedal and the brake pedal are realized by logical judgment. This article sets the following rules: (1) if there is a brake pedal signal, the output of the fuzzy controller recognition results; (2) if there is a brake pedal signal, and the throttle pedal signal is greater than the brake pedal signal, the recognition result is normal acceleration; (3) if there is a brake pedal signal, and the throttle pedal signal is less than the brake pedal signal, the recognition result is braking. The driving intention recognition process is shown in Figure 7.

## 4. Simulation Research

**Figure 8:**
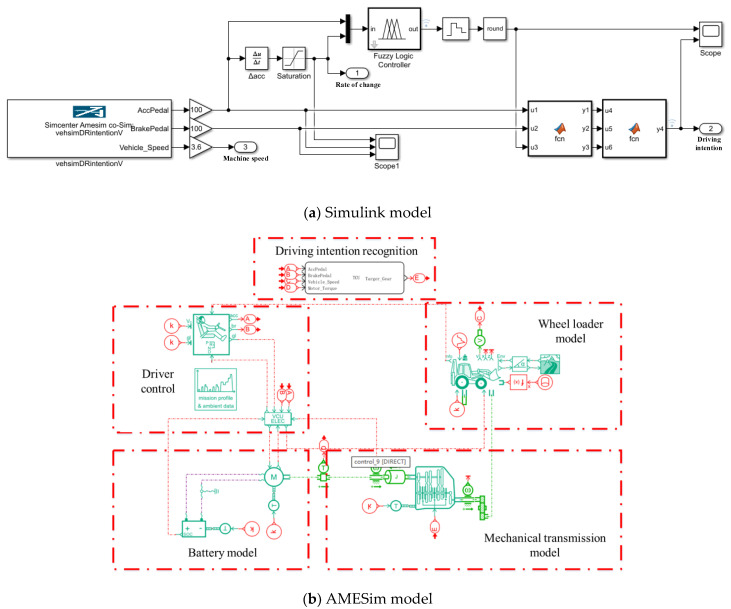
Driving intention recognition simulation model.

In this paper, a 5 t electric wheel loader is studied. The basic parameters of the wheel loader are shown in Table 6. Using Matlab/Simulink (R2018 a) and AMESim co-simulation, the driving intention recognition model is built in Simulink, and the electric wheel loader vehicle model is built in AMESim. Since the vehicle model built in AMESim can better simulate the driver’s operation of the accelerator pedal and the brake pedal during actual driving, the signal in the AMESim simulation process is selected as the input signal for acceleration intention recognition. The recognition model is shown in Figure 8.

**Figure 9:**
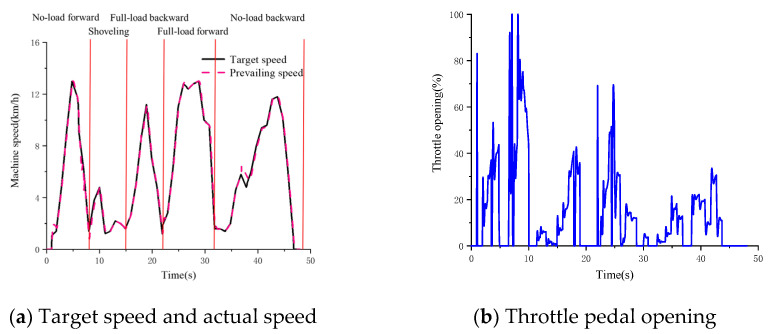

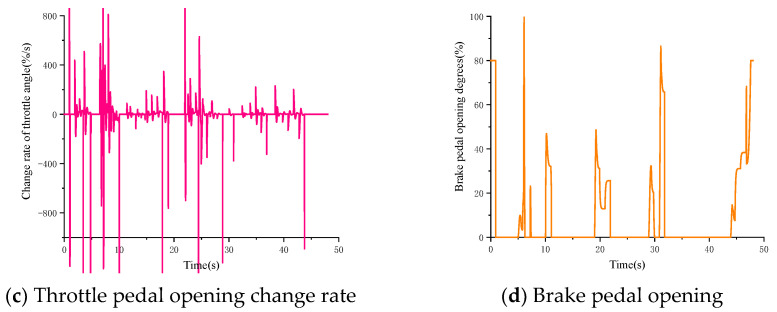
Driving intention recognition input.

The ‘V’ working condition of the wheel loader is selected to simulate and verify the driving intention. The actual speed, throttle pedal opening and brake pedal opening of the identified input are derived from the AMESim simulation model. The change rate of throttle pedal opening is obtained by time and throttle pedal opening. All inputs are shown in Figure 9.

**Figure 10:**
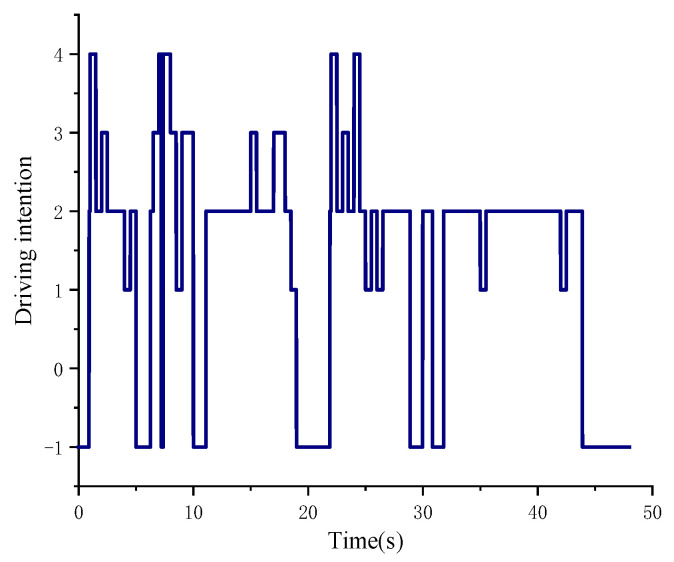
Driving intention recognition results.

The driving intention recognition results are shown in Figure 10.

The driving intention recognition result number corresponds to the following: 4: rapid acceleration; 3: normal acceleration; 2: maintenance; 1: deceleration; −1: braking.

During the operation of the wheel loader in the whole ‘V’-type working condition, the driver will frequently manipulate the accelerator pedal and the brake pedal. Therefore, in order to reflect the driver’s intention in time and effectively, the recognition period set in the simulation of this paper is 0.5 s. The results of driving intention recognition are analyzed by combining Figure 9 and Figure 10. When the wheel loader is just starting, the throttle pedal opening increases rapidly, and the intention recognition result is rapid acceleration, which is more accurate. In the range of 5~6.5 s, the accelerator pedal is quickly loosened and the brake signal appears. During this period, the driving intention is first identified as deceleration, and the recognition result is brake when there is a brake signal, which is more accurate. The interval of 6.5~10 s is the shoveling stage, and the recognition result is rapid acceleration, which is more accurate. The throttle opening of 25~26 s is slowly loosened, the vehicle speed is reduced and the recognition result is deceleration, which is more accurate. The throttle opening in the 35~42 s interval is small and the change rate of the throttle opening is small. The recognition result is maintained and more accurate. In summary, the method based on fuzzy control can accurately identify the driver’s intention.

## 5. Experimental Research

**Figure 11:**
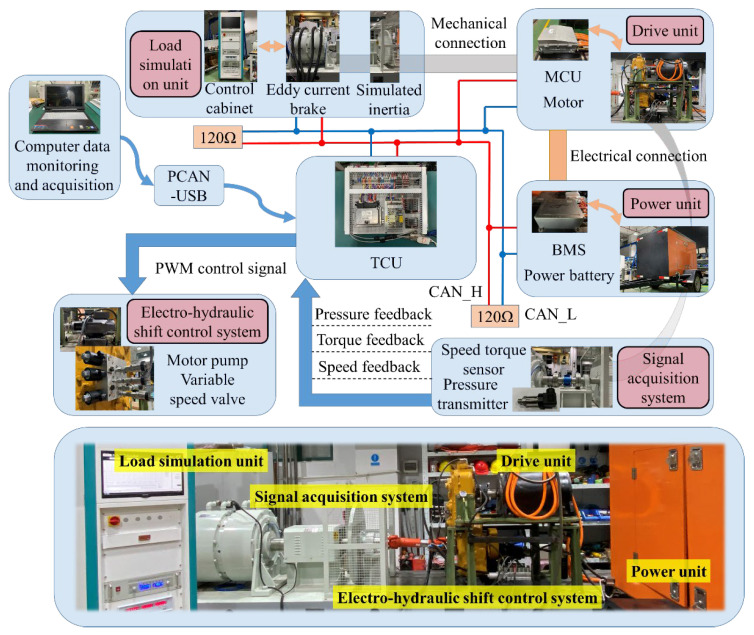
Comprehensive test platform for electromechanical hydraulic transmission system of electric wheel loader.

In order to further verify its effectiveness, a simulation test bench for the automatic transmission system of electric wheel loaders is built and experimental research is carried out. The experimental bench is shown in Figure 11. The components of the test bench are consistent with the actual wheel loader, and are mainly composed of a power unit, drive unit, signal acquisition unit, load simulation unit, electro-hydraulic shift control system and so on. The power unit is composed of power batteries to provide high-voltage electric energy for the drive motor. The drive unit is mainly composed of a drive motor, motor controller and transmission. The signal acquisition unit transmits the real-time signal of each sensor through CAN communication. The load simulation unit is mainly composed of an inertia disk, eddy current brake and control cabinet to simulate the external load of the vehicle driving and working conditions.

The transmission control unit (TCU) controller selected is the TTC60, featuring an Infineon (Munich, Germany) XC2287 CPU. This controller is equipped with multiple independently programmable general-purpose I/O interfaces, multiple CAN and RS232 communication interfaces, as well as multiple PWM outputs, which facilitate the implementation of a multi-functional test platform.

(1) Operator Input Signals: These include signals from the electronic accelerator pedal, electronic brake pedal, automatic shift switch, gear selection switch, drive mode selection switch, cruise mode switch, brake switch, regenerative brake switch, safety lock switch, power-on/off switch, cooling system switch, emergency switch, and others.
(2) Detection Input Signals: These include analog signals such as the transmission output shaft speed, output shaft torque, oil pressure, and oil temperature.
(3) Control Output Signals: These include solenoid valve control signals for shifting and safety locking.
(4) Status Monitoring Signals: These include information on speed, torque, oil pressure, oil temperature, gear position, and faults.
(5) Communication Interfaces: These include an RS232 serial communication interface for controller development and debugging, a CAN communication interface, and CAN communication interfaces for various components on the platform that simulate the CAN communication of a complete vehicle.

In the experimental process, the driver is required to drive according to the maintenance–acceleration–maintenance–small acceleration–deceleration–acceleration–braking intention in advance. The driver simulates driving by manipulating the accelerator pedal and the brake pedal. The braking force corresponding to the brake pedal is simulated by applying a load to the eddy current brake. The test results are shown in Figure 11, where 1 represents braking, 0 represents no intention to output, 1 represents deceleration, 2 represents maintenance, 3 represents normal acceleration, and 4 represents rapid acceleration.

**Figure 12:**
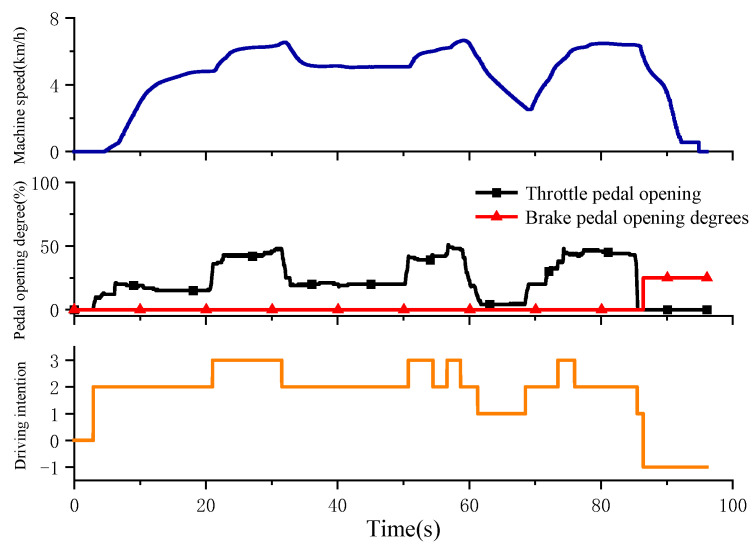
Driving intention recognition test results.

As shown in Figure 12, the driving intention test results are the speed, the throttle pedal and brake pedal opening and the driving intention recognition results, respectively. The slope of the throttle opening curve can reflect the magnitude of the change rate of the throttle opening. From the results, it can be concluded that from 3 s to 20 s, starting with a small throttle, the change rate of throttle opening and throttle opening is small, which is identified as maintenance intention. During the period from 20 s to 32 s, the accelerator pedal increases rapidly, which is identified as acceleration intention. During the period from 32 s to 51 s, the current vehicle speed is maintained, which is identified as maintenance intention. During the period from 51 s to 60 s, a small amount of acceleration is carried out, the throttle opening increases, and the recognition result is acceleration. During the period from 60 s to 69 s, the accelerator pedal is loosened, the vehicle speed decreases and the recognition result is deceleration intention. During the period from 69 s to 85 s, the accelerator pedal increases slowly, which is identified as maintenance in the slow stage, and normal acceleration is identified at the moment of rapid increase. During the period from 85 s to 86 s, the accelerator pedal is quickly loosened and identified as the deceleration intention, and then the brake pedal signal appears and is identified as the braking intention. According to the above analysis, the fuzzy controller can more sensitively identify the current intention according to the driver’s manipulation, and the recognition result is basically consistent with the specified test intention, so it shows that the method can accurately identify the driver’s intention.

## 6. Conclusions

The wheel loader’s working process shifts frequently, and automatic shifting can effectively improve the driver’s driving safety and economy. The driving intention is an important input judgment condition for achieving efficient automatic shift. However, the current methods of vehicle driving intention recognition mainly focus on passenger cars. In this paper, aiming at the electric wheel loader, according to the characteristics of wheel loader working conditions, a fuzzy control driving intention recognition method is proposed. The driving intention of the wheel loader is divided into rapid acceleration, normal acceleration, maintenance, deceleration and braking intention. The throttle, throttle change rate and braking signal are selected as the input parameters of recognition, and the fuzzy rules of driver intention recognition are constructed to obtain the driver’s driving intention for wheel loader driving process. Simulation and experimental studies are carried out, respectively. The results show that the driving intention recognition model based on fuzzy control can accurately identify the driving intention. It can provide effective input for the automatic gear shifting control of electric wheel loaders.

## Figures and Tables

**Table 1:** The classification and characteristics of accelerated intention.

Driving Intention	Classification	Feature
Accelerated intention	Rapid acceleration	The throttle pedal opening and the rate of change is very large.
	Normal acceleration	The throttle opening change rate is moderate.
	Maintenance	The change rate of throttle opening is small or constant.

**Table 2:** Classification and characteristics of deceleration and braking intention.

Driving Intention	Classification	Feature
Deceleration, braking intention	Deceleration	The throttle pedal opening decreases and the throttle opening change rate is negative.
	Braking	The throttle pedal opening decreases rapidly and the brake pedal opening is positive.

**Table 3:** Input variable membership function.

Fuzzy Variable	Universe of Discourse	Linguistic Value	Membership Function
Throttle pedal opening	0~20%	Zero ZO	Trapezium
	10~40%	Positive small PS	Triangle
	20~70%	Positive medium PM	Triangle
	40~100%	Positive big PB	Trapezium
Change rate of throttle angle	−1000~−75%/s	Negative big NB	Trapezium
	−125~−25%/s	Negative medium NM	Triangle
	−75~0%/s	Negative small NS	Triangle
	−10~10%/s	Zero ZO	Triangle
	0~75%/s	Positive small PS	Triangle
	25~125%/s	Positive medium PM	Triangle
	75~800%/s	Positive big PB	Trapezium

**Table 4:** Output variable membership function.

Fuzzy Variable	Linguistic Value	Numbering	Membership Function
Driver’s intention	Braking	−1	Single value
	Deceleration	1	Single value
	Maintenance	2	Single value
	Normal acceleration	3	Single value
	Rapid acceleration	4	Single value

**Table 5:** Driver intention recognition fuzzy rules.

Driver’s Intention		Throttle Pedal Opening			
		Zero	Small	Medium	Big
Throttle pedal opening rate of change	Positive big PB	Acceleration	Acceleration	Rapid acceleration	Rapid acceleration
	Positive medium PM	Acceleration	Acceleration	Acceleration	Rapid acceleration
	Positive small PS	Maintenance	Maintenance	Acceleration	Acceleration
	Zero ZO	Maintenance	Maintenance	Maintenance	Acceleration
	Negative small NS	Maintenance	Maintenance	Deceleration	Deceleration
	Negative medium NM	Deceleration	Deceleration	Deceleration	Deceleration
	Negative big NB	Deceleration	Deceleration	Deceleration	Deceleration

**Table 6:** Basic parameters of the loader.

Parameters	Unit	Value
Size (length × width × height)	mm	8130 × 2880 × 3450
Mass	kg	17,400
Rated load	kg	5000
Maximum speed	km/h	≥40
Maximum traction torque	kN	155 ± 5
Maximum breakout force	kN	167
Type of drive axle	-	Four-wheel drive
Drive axle ratio	-	4.375
Wheel-end ratio	-	5.2

## Data Availability

The data is available on the request.
